# CROSS-GENERATIONAL UNDERSTANDING OF AGEISM AND ITS IMPACT ON PERSONAL-PUBLIC HEALTH

**DOI:** 10.1093/geroni/igz038.3265

**Published:** 2019-11-08

**Authors:** Diana Mayo, Thomas M Meuser, Regula H Robnett

**Affiliations:** 1 University of New England, Biddeford, Maine, United States; 2 University of New England, Portland, Maine, United States

## Abstract

In 2016, the World Health Organization (WHO) declared a call to combat ageism, labeling it “pervasive” and having “profound consequences on older adults’ health and well being.” This study explored generational differences in understanding the WHO’s definition of ageism, between baby boomers (ages 65-72) and silent generation members (ages 78-85), as well as the perceived impact on personal and public health outcomes. A focus group protocol built around the WHO framing of ageism was administered to boomer (n=18) and silent generation members (n=11). Discussion was transcribed, reviewed in depth by each research team member, and themes were extracted by consensus. Members of both cohorts initially denied effects of ageism, stating that they reject discriminatory behavior; later sharing explicit examples of ageism’s negative impact on their lives. Boomers conflated the words “ageism” and “aging”, perhaps implying a lack of awareness of the terms and the issues as presented by WHO. A central finding was that older adults in both groups experienced economic and health care disparities due to their age. In both groups, perceived perpetrators of discriminatory behavior were found in various environments including places of employment, healthcare sites, restaurants, public transportation, retirement communities, and at home among family and care services. Our results are critical to understanding what environments to target for public health intervention efforts, which will include establishing future education and training for people of all ages to help society learn about ageism, and to advocate for inclusive and equitable treatment of older adults in the community.

